# Association and predictive value of sarcopenic obesity for the prognosis of lung cancer patients receiving immune checkpoint inhibitors

**DOI:** 10.3389/fnut.2026.1687912

**Published:** 2026-03-11

**Authors:** Tingting Yang, Wen Wang, Yan Qiao, Ke Guan, Weixiang Wang, Yang Yang, Yongchun Chen, Lijun Shen

**Affiliations:** 1 Department of Nutrition, Henan Provincial People’s Hospital and Zhengzhou University People’s Hospital, Zhengzhou, Henan, China; 2 Department of Infection Management, Tianjin Union Medicine Center, The First Affiliated Hospital of Nankai University, Tianjin, China; 3 Department of Respiratory and Critical Care Medicine, Henan Provincial People’s Hospital, Zhengzhou University People’s Hospital, Zhengzhou, Henan, China

**Keywords:** sarcopenic, obesity, lung cancer, ICIs, ECOG-PS

## Abstract

**Background:**

Sarcopenic obesity (SO) has been established as a reliable predictor of prognosis for several cancer types; however, its role in the prognosis of lung cancer patients receiving immune checkpoint inhibitors (ICIs) remains unclear. This study aimed to explore the potential predictive value of SO on survival outcomes in lung cancer patients undergoing ICI therapy.

**Methods:**

From May 2018 to October 2020, lung cancer patients who received immunotherapy at a tertiary hospital in Henan Province were retrospectively evaluated using data from the electronic medical record system. Data on demographic characteristics, biochemical markers, current illnesses and treatments, and nutrition-related information were documented. Sarcopenia was evaluated using the skeletal muscle index (SMI) (cm^2^/m^2^), which was calculated by measuring the muscle mass area from a cross-sectional CT image at the L3 vertebra level prior to immunotherapy.

**Results:**

The 119 participants were divided into four groups: control, sarcopenia, obesity, and SO. Among these participants, 15.13% were diagnosed with SO. The results demonstrated that for 3-year survival rates, patients with SO had the highest mortality rate (with a median survival of 22.55 months), followed by those with sarcopenia alone (median survival: 28.29 months) and those with obesity alone (median survival: 28.99 months) (*p* < 0.05). Multivariate Cox regression analysis indicated that SO (*HR* = 3.479, 95% *CI* = 1.374–8.814), creatinine level (*HR* = 0.963, 95% *CI* = 0.936–0.990), receiving ICIs as second-line therapy (*HR* = 4.274, 95% *CI* = 1.941–9.411), receiving ICIs as third-line or later therapy (*HR* = 2.980, 95% *CI* = 1.169–7.597), and an Eastern Cooperative Oncology Group performance status (ECOG-PS) score of ≥3 (*HR* = 5.274, 95% *CI* = 2.670–10.418) are independent factors associated with reduced 3-year survival time.

**Conclusion:**

SO is an independent prognostic factor for lung cancer patients receiving immunotherapy. Early identification and targeted management of sarcopenic obesity are crucial for optimizing treatment strategies and improving survival outcomes in ICI-treated lung cancer patients.

## 1 Introduction

Lung cancer, a malignant tumor with high incidence and mortality rates worldwide, poses a serious threat to human health. Epidemiological data indicate that lung cancer exhibits the highest incidence among male malignancies and ranks second in female populations (1). Notably, it has the highest mortality rate across all cancer types. According to the 2020 global cancer incidence and mortality statistics, lung cancer accounted for 11.4% of all cancer cases, making it the second most common malignancy after breast cancer, and both the incidence and mortality rates of lung cancer are experiencing an upward trend worldwide (1, 2).

The advent of immune checkpoint inhibitors (ICIs) has revolutionized treatment paradigms for lung cancer, offering remarkable survival benefits and improvements in quality-of-life in clinical practice (3, 4). However, significant variability in treatment responses among patients remains a challenge. Increasing evidence suggests that intrinsic patient factors—particularly nutritional status and body composition—may critically modulate the efficacy of immunotherapy (5). Among these, sarcopenic obesity (SO), a pathophysiological condition characterized by concurrent sarcopenia and excess fat accumulation (6, 7), has emerged as a potential determinant of therapeutic outcomes. Previous studies have established SO as a robust predictor of cancer prognosis. Liu et al. (8) conducted a prospective cohort study involving 6,790 patients with solid tumors and found that SO was associated with worse overall survival (hazard ratio, *HR* = 1.54), reduced quality of life, and an increased risk of ICU admission. Gao et al. (9) performed a comprehensive systematic review and meta-analysis encompassing 38 studies involving 10,004 patients with cancer. This analysis revealed an aggregate prevalence of SO at 20% and significant correlations with poorer overall survival (*HR* = 1.83), reduced recurrence-free survival (*HR* = 2.10), increased surgical complications (*HR* = 3.01), and longer hospital stays (*HR* = 5.69).

However, evidence regarding the prognostic impact of SO on lung cancer patients undergoing ICI therapy remains limited. Although SO has been recognized as a prognostic factor in various cancers, its specific impact on outcomes following ICI therapy in lung cancer patients has not been fully elucidated. To address this knowledge gap, we designed a retrospective cohort study to systematically evaluate the effects of both sarcopenia and SO on treatment outcomes in ICI-treated lung cancer patients.

## 2 Materials and methods

### 2.1 Study participants

This retrospective study analyzed data obtained from lung cancer patients who received ICIs at a tertiary hospital in Henan Province, China. The inclusion criteria were as follows: (1) age ≥18 years; (2) pathologically confirmed lung cancer; (3) receipt of at least two cycles of ICI therapy; (4) availability of an abdominal CT scan performed within 1 week prior to the initiation of ICI therapy; and (5) complete clinical, laboratory, and imaging data. The exclusion criteria included the following: (1) a prior history of immunotherapy for lung cancer, (2) concurrent primary malignancies at other sites, (3) the use of hormonal therapy, or (4) absence of imaging of the third lumbar vertebra (L3) level in CT scans. The study protocol was approved by the Ethics Committee of Henan Provincial People’s Hospital.

### 2.2 Data collection and follow-up

From May 2018 to October 2020, 146 lung cancer patients who met the predefined inclusion and exclusion criteria and received ICI therapy were identified in the hospital’s electronic medical record system for inclusion in the study. Baseline clinical data were retrospectively extracted from medical records, including age, gender, height, weight, body mass index (BMI), smoking status, tumor pathology, tumor stage (according to the 8th edition of the AJCC), surgical history, metastasis status, and laboratory results (including neutrophil, lymphocyte, and monocyte counts and hemoglobin, albumin, creatinine, and cystatin levels), line of ICI therapy, and whether the therapy was combined with chemotherapy, with radiotherapy (RT)/targeted therapy. Other data extracted included the patient’s Eastern Cooperative Oncology Group Performance Status (ECOG-PS), Nutritional Risk Screening 2002 (NRS2002), Global Leadership Initiative on Malnutrition (GLIM), and the Prognostic Nutritional Index (PNI). Overall survival (OS) was defined as the time from the initiation of immunotherapy until death from any cause or the last follow-up (September 2023), with follow-up limited to a maximum of 3 years. Ultimately, 119 patients with complete information were included in the analysis.

### 2.3 Diagnostic criteria and classification of sarcopenia

The skeletal muscle area (SMA) at the L3 level on CT scans was used as a reliable indicator of whole-body skeletal muscle mass. All participants underwent CT scans within 1 week before immunotherapy. A quantitative analysis was performed using software developed by Shen et al.^1^ The specific procedures included: (1) SMA segmentation using Hounsfield unit (HU) thresholds (−29 to 150) and (2) automated calculation of the SMA using a convolutional neural network (CNN), which is a type of deep learning algorithm. The skeletal muscle index (SMI) was calculated by dividing the SMA (cm^2^) by the square of height (m^2^) (SMI = SMA/height^2^). Sarcopenia was defined as an SMI < 43 cm^2^/m^2^ for men with a BMI < 25 kg/m^2^, an SMI < 53 cm^2^/m^2^ for men with a BMI ≥ 25 kg/m^2^, and an SMI < 38 cm^2^/m^2^ for women (10). This classification has been extensively utilized in oncological research and demonstrates significant prognostic value for cancer patients (11, 12). The visceral fat area (VFA) was manually measured at the umbilical level using HU ranges of −150 to −50. All measurements were independently performed by two trained operators.

Patients were classified into four body composition subtypes: control (BMI < 24.0 kg/m^2^ without sarcopenia), sarcopenic (BMI < 24.0 kg/m^2^ with sarcopenia), obese (BMI ≥ 24.0 kg/m^2^ without sarcopenia), and SO (BMI ≥ 24.0 kg/m^2^ with sarcopenia).

### 2.4 Statistical analysis

Continuous variables with a normal distribution were presented as the mean ± standard deviation, while those with a non-normal distribution were expressed as the median (25th and 75th percentiles). Comparisons between groups for continuous variables were conducted using the Kruskal–Wallis H test or analysis of variance (ANOVA). Categorical variables were presented as frequencies (percentages). Differences among categorical variables were assessed using Pearson’s chi-squared test. The Kaplan–Meier method and log-rank testing were used to analyze the association between body composition characteristics and 3-year overall survival (OS). Univariable and multivariable Cox regression analyses were performed to identify independent prognostic factors influencing OS in lung cancer patients receiving immunotherapy. A two-sided *p*-value of <0.05 was considered statistically significant. The data were analyzed using SPSS 22.0.

## 3 Results

### 3.1 Baseline characteristics of the study population

Based on the inclusion and exclusion criteria, 146 hospitalized lung cancer patients receiving immunotherapy were initially enrolled. After excluding 9 cases with missing data on sarcopenia, 15 cases lost to follow-up, and 3 cases lacking both sarcopenia data and follow-up information, a total of 119 patients (81.5% of the original cohort) met all eligibility requirements and were included in the final analysis. Control (*n* = 46; 38.65%); sarcopenia (*n* = 23; 19.33%); obesity (*n* = 32; 26.89%); and SO (*n* = 18; 15.13%). The final cohort consisted of 79 men (66.40%) and 40 women (33.60%), with a mean age of 61.22 ± 11.56 years and a mean BMI of 23.33 ± 3.12 kg/m^2^. Differences were observed between the SO group and the other groups in terms of height, weight, BMI, visceral fat area (VFA), SMI, lymphocyte count, and hemoglobin levels (*p* < 0.05) (Table 1).

**Table 1:** Baseline characteristic of patients.

Variables	Total (*n* = 119)	Control (*n* = 46)	Sarcopenia (*n* = 23)	Sarcopenic obesity (*n* = 18)	Obesity (*n* = 32)	*p*-value
Demographic characteristics						
Gender						0.052
Male	79 (66.40)	30 (65.20)	11 (47.80)	16 (88.90)	22 (68.80)	
Female	40 (33.60)	16 (34.80)	12 (52.20)	2 (11.10)	10 (31.20)	
Age (year)	61.22 ± 11.56	60.11 ± 10.86	64.87 ± 10.33	64.39 ± 8.10	58.41 ± 14.16	0.112
Height (cm)	165.23 ± 8.28	163.91 ± 8.75	162.83 ± 6.75	171.06 ± 7.70	165.56 ± 7.58	0.006
Weight (kg)	64.12 ± 11.69	59.00 ± 7.17	53.39 ± 7.17	76.72 ± 9.36	72.09 ± 8.36	<0.001
BMI (kg/m^2^)	23.33 ± 3.12	22.33 (21.15, 23.23)	20.11 ± 2.12	25.51 (24.94, 27.26)	25.71 (24.44, 27.68)	<0.001
VFA (cm^2^)	120.34 ± 72.59	88.06 ± 50.67	65.69 ± 48.79	164.20 ± 68.71	181.37 ± 57.68	<0.001
SMI (cm^2^/m^2^)	46.53 ± 8.20	47.71 ± 5.36	36.34 ± 3.53	44.88 ± 4.87	53.11 ± 8.17	<0.001
Smoking						0.842
Yes	40 (33.60)	16 (34.80)	6 (26.10)	6 (33.30)	12 (37.50)	
No	79 (66.40)	30 (65.20)	17 (73.90)	12 (66.70)	20 (62.50)	
Biochemical markers						
Neutrophils (×10^9^/L)	4.21 (2.95, 5.56)	3.52 (2.83, 6.14)	3.67 (2.95, 5.12)	5.06 ± 1.97	4.55 (2.98, 5.33)	0.466
Lymphocyte (×10^9^/L)	1.24 (0.89, 1.83)	1.51 ± 0.63	1.09 (0.84,1.50)	1.02 (0.87,1.78)	1.24 (0.97, 1.73)	0.356
Monocyte count (×10^9^/L)	0.43 (0.32,0.57)	0.45 (0.27,0.57)	0.40 (0.32,0.58)	0.50 ± 0.21	0.43 (0.33, 0.52)	0.356
Hemoglobin (g/L)	123.95 ± 19.68	125.51 ± 20.27	113.26 ± 16.04	122.17 ± 18.45	130.41 ± 19.39	0.012
Albumin (g/L)	39.90 (35.80, 43.10)	40.40 (35.98, 43.43)	37.93 ± 5.59	38.45 ± 5.17	40.73 ± 4.18	0.857
Creatinine (μmol/L)	59.00 (50.00, 68.00)	60.50 (53.75, 69.75)	55.43 ± 15.81	64.00 (56.50, 69.00)	58.28 ± 11.37	0.170
Cystatin (mg/L)	0.93 (0.85, 1.05)	0.91 (0.84, 1.02)	1.01 ± 0.19	1.04 ± 0.21	0.90 ± 0.13	0.122
Current disease and treatment						
Pathologist classification						0.992
Small cell carcinoma	11 (9.20)	4 (8.70)	2 (8.70)	2 (11.10)	3 (9.40)	
Non-small cell carcinoma	108 (90.80)	42 (91.30)	21 (91.30)	16 (88.90)	29 (90.60)	
Stages						0.628
II/III	30 (25.20)	9 (19.60)	7 (30.40)	6 (33.30)	8 (25.00)	
IV	89 (74.80)	37 (80.40)	16 (69.60)	12 (66.70)	24 (75.00)	
Surgery						0.341
Yes	13 (10.90)	8 (17.40)	2 (8.70)	1 (5.60)	2 (6.20)	
No	106 (89.10)	38 (82.60)	21 (91.30)	17 (94.40)	30 (93.80)	
Metastasis						0.169
Yes	18 (15.10)	10 (21.70)	4 (17.40)	0 (0.00)	4 (12.50)	
No	101 (84.90)	36 (78.30)	19 (82.60)	18 (100.00)	28 (87.50)	
Line of ICIs therapy						0.226
1L	62 (52.10)	30 (65.20)	9 (39.10)	8 (44.40)	15 (46.90)	
2L	37 (31.10)	13 (28.30)	9 (39.10)	6 (33.30)	9 (28.10)	
≥3L	20 (15.80)	3 (6.50)	5 (21.80)	4 (22.30)	8 (25.00)	
Combination with chemotherapy						0.177
Yes	91 (76.50)	39 (84.80)	14 (60.90)	14 (77.80)	24 (75.00)	
No	28 (23.50)	7 (15.20)	9 (39.10)	4 (22.20)	8 (25.00)	
Combination with RT/targeted therapy						0.564
Yes	82 (68.90)	33 (71.70)	13 (56.60)	13 (72.70)	23 (71.90)	
No	37 (31.10)	13 (28.30)	10 (43.50)	5 (27.80)	9 (28.10)	
ECOG-PS						0.268
0–2	91 (76.50)	37 (80.40)	14 (60.90)	14 (77.80)	26 (81.30)	
3–4	28 (23.50)	9 (19.60)	9 (39.10)	4 (22.20)	6 (18.80)	
Nutrition-related information						
NRS2002						0.362
<3	82 (68.90)	29 (63.00)	15 (65.20)	12 (66.70)	26 (81.30)	
≥3	37 (31.10)	17 (37.00)	8 (34.80)	6 (33.30)	6 (18.70)	
GLIM severity grading						0.015
No	93 (78.20)	36 (78.20)	15 (65.30)	14 (77.80)	28 (87.50)	
Moderate	13 (10.90)	5 (10.90)	1 (4.30)	4 (22.20)	3 (9.40)	
Severe	13 (10.90)	5 (10.90)	7 (30.40)	0 (0.00)	1 (3.10)	
PNI	46.42 ± 6.42	46.93 ± 7.11	44.11 ± 6.94	45.55 ± 4.32	47.82 ± 5.67	0.163

### 3.2 Impact of sarcopenic obesity on survival time

**Figure 1:**
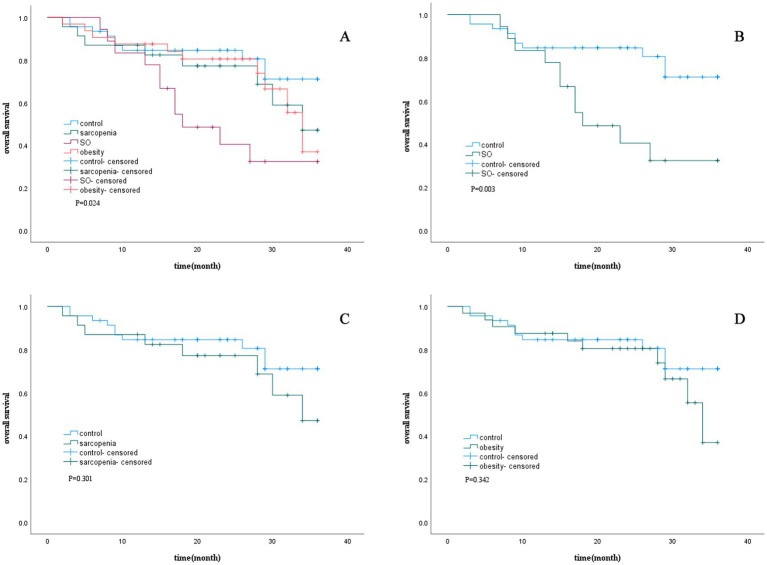
Survival curve according to sarcopenic status. **(A)** Kaplan–Meier curves for 3-year overall survival in patients with obesity, sarcopenia, SO and control. **(B)** Kaplan–Meier curves for 3-year overall survival in patients with and without SO. **(C)** Kaplan–Meier curves for 3-year overall survival in patients with and without sarcopenia. **(D)** Kaplan–Meier curves for 3-year overall survival in patients with and without obesity. SO, sarcopenic obesity.

The impact of SO on survival time in lung cancer patients was analyzed using Kaplan–Meier curves (Figure 1). The results demonstrated that for 3-year survival rates, patients with SO had the highest mortality rate (median survival: 22.55 months), followed by those with sarcopenia alone (median survival: 28.29 months) and those with obesity alone (median survival: 28.99 months). In contrast, the control group exhibited the lowest mortality rate (median survival: 30.44 months). Significant differences were observed between the survival curves (*p* = 0.024).

Further survival analyses, stratified by the presence of sarcopenia, SO, or obesity, showed that SO was associated with significantly shorter survival times compared to the control group, with statistically significant differences in the survival curves (*p* < 0.05).

### 3.3 Impact of sarcopenic obesity on survival time

The results of univariable and multivariable Cox regression analyses are presented in Table 2. Univariate Cox regression analysis of 3-year survival in lung cancer patients receiving immunotherapy identified several significant factors as follows: SO (*HR* = 3.479, 95% CI = 1.470–8.237), lymphocyte count (*HR* = 0.440, 95% CI = 0.244–0.795), hemoglobin level (*HR* = 0.977, 95% CI = 0.961–0.993), albumin level (*HR* = 0.917, 95% CI = 0.868–0.970), line of ICI therapy (2 L: *HR* = 3.683, 95% CI = 1.731–7.838; ≥3 L: *HR* = 3.475, 95% CI = 1.479–8.215), combination with chemotherapy (*HR* = 0.460, 95% CI = 0.231–0.918), an ECOG-PS ≥ 3 (*HR* = 3.959, 95% CI = 2.091–7.495), and the prognostic nutritional index (PNI) (*HR* = 0.906, 95% CI = 0.863–0.952). The multivariate analysis identified the following independent prognostic factors: SO (*HR* = 3.479, 95% CI = 1.374–8.814), creatinine level (*HR* = 0.963, 95% *CI* = 0.936–0.990), receiving ICIs as second-line therapy (*HR* = 4.274, 95% CI = 1.941–9.411), receiving ICIs as third-line or later therapy (*HR* = 2.980, 95% CI = 1.169–7.597), and an ECOG-PS ≥ 3 (*HR* = 5.274, 95% CI = 2.670–10.418).

**Table 2:** Univariate cox regression analysis of survival time.

Variables	Univariable model			Multivariable Model		
	*HR*	95%*CI*	*p*	*HR*	95%*CI*	*p*
Gender (ref. male)	1.054	0.541–2.054	0.876			
Age (year)	1.021	0.991–1.050	0.168			
VFA (cm^2^)	1.002	0.997–1.006	0.423			
Smoking (ref. No)	1.247	0.653–2.380	0.504			
Sarcopenic status (ref. Control)						
Obesity	1.491	0.620–3.586	0.372	0.976	0.406–2.533	0.976
Sarcopenia	1.632	0.643–4.140	0.303	0.578	0.204–1.641	0.303
Sarcopenic obesity	3.479	1.470–8.237	0.005	3.479	1.374–8.814	0.009
Biochemical markers						
Neutrophils (×10^9^/L)	1.043	0.928–1.173	0.478			
Lymphocyte (×10^9^/L)	0.440	0.244–0.795	0.006			
Monocyte count (×10^9^/L)	0.993	0.500–1.969	0.983			
Hemoglobin (g/L)	0.977	0.961–0.993	0.005			
Albumin (g/L)	0.917	0.868–0.970	0.002			
Creatinine (μmol/L)	0.978	0.955–1.002	0.078	0.963	0.936–0.990	0.008
Cystatin (mg/L)	0.848	0.155–4.641	0.849			
Current disease and treatment						
Pathologist classification (ref. Small cell carcinoma)						
Non-small cell carcinoma	2.592	0.623–10.782	0.190			
Stages (ref. III or II)						
IV	2.090	0.917–4.766	0.080			
Surgery (ref. no)						
Yes	0.472	0.113–1.965	0.302			
Metastasis (ref. no)						
Yes	1.622	0.745–3.534	0.223			
Line of ICIs therapy (ref.1 L)						
2 L	3.683	1.731–7.838	<0.001	4.274	1.941–9.411	0.001
≥3	3.475	1.479–8.215	0.005	2.980	1.169–7.597	0.022
Combination with chemotherapy (ref. No)						
Yes	0.460	0.231–0.918	0.028			
Combination with RT/targeted therapy (ref. No)						
Yes	1.280	0.623–2.629	0.501			
ECOG-PS (ref. 0–2)						
3–4	3.959	2.091–7.495	<0.001	5.274	2.670–10.418	<0.001
Nutrition-related information						
NRS2002 (ref. <3)						
≥3	1.425	0.737–2.756	0.292			
GLIM (ref. No)						
Moderate	1.503	0.579–3.904	0.402			
Severe	1.714	0.709–4.143	0.231			
PNI	0.906	0.863–0.952	<0.001			

## 4 Discussion

This study used a retrospective cohort design to investigate the influencing factors of survival time in ICI-treated lung cancer patients and explore the predictive value of sarcopenia and SO on the survival period after receiving immunotherapy. Cox regression analyses revealed that SO, an ECOG-PS ≥ 3, line of ICI therapy ≥2 L, and low creatinine levels were independent factors associated with reduced survival time in lung cancer patients. Kaplan–Meier curve analysis visually presented the survival differences among lung cancer patients across different groups. The results showed that the 3-year survival rate of immunotherapy in lung cancer patients with SO was the lowest, followed by the sarcopenic and obesity groups. In contrast, the control group exhibited the most favorable outcomes. These findings highlight the critical need for routine assessment and targeted intervention for SO in lung cancer patients receiving immunotherapy.

A multivariable Cox regression analysis further clarified the independent prognostic factors associated with the 3-year survival outcomes of lung cancer patients. The results indicated that even after multivariate adjustment, SO (*HR* = 3.479, 95% CI = 1.374–8.814) continued to have a consistently negative impact on patient survival, reflecting a significantly higher risk ratio. Therefore, it plays a crucial role in clinical prognosis assessment. Our findings align with previous conclusions that cancer patients who develop SO tend to have a poorer prognosis. A systematic review and meta-analysis of 38 observational studies on the prevalence and prognostic significance of SO in cancer patients revealed that SO individuals had a 1.83-fold increased mortality risk (*RR* = 1.83, 95% CI: 1.41–2.38) compared to non-sarcopenic obese patients (9). A cohort study conducted by Li Y et al. (13) identified that SO was a significant predictor of survival outcomes in hepatocellular carcinoma (HCC) patients undergoing transarterial chemoembolization (TACE). Previous research on SO and survival outcomes in lung cancer patients has been relatively limited, with the majority of studies focusing primarily on the prognostic impact of sarcopenia alone in this population. Bolte et al. (14) found that sarcopenia independently predicted worse overall survival (OS) in non-small cell lung cancer (NSCLC) patients (*HR* = 2.12), with sarcopenic patients having a median OS of 9.1 months compared to 22.3 months in non-sarcopenic patients. A systematic review conducted by Lin et al. (15) confirmed that sarcopenia is common among lung cancer patients and is linked to a higher incidence of postoperative complications, shorter progression-free survival after immunotherapy, and reduced OS. Moreover, sarcopenic patients had significantly higher mortality rates, regardless of cancer stage or treatment, reinforcing its negative impact on survival outcomes.

The mechanism by which SO influences the prognosis of lung cancer patients receiving immunotherapy remains unclear. Several studies have indicated that muscle loss leads to a reduction in the secretion of certain immunomodulatory factors by muscle cells, such as interleukin-7 (IL-7) and interleukin-15 (IL-15). When the secretion of these cytokines is insufficient, both the quantity and function of T cells are affected, resulting in a weakened ability of the body’s immune surveillance and immune response against tumor cells (16–18). In the context of obesity, there is an excessive accumulation of adipose tissue in the body, and adipocytes secrete large quantities of inflammatory cytokines, such as tumor necrosis factor-alpha (TNF-*α*) and interleukin-6 (IL-6). These inflammatory cytokines can inhibit the proliferation and activation of T cells and interfere with the normal functioning of the immune system (19–21). In addition, obesity can also lead to metabolic disorders in immune cells, affecting the energy supply and functional performance of T cells. This impairs their ability to be effectively activated and exert their immune-killing function against tumor cells, thereby creating favorable conditions for the growth and escape of tumors (22, 23). The complex interaction between SO and the tumor microenvironment has a significant impact on the growth of lung cancer and the efficacy of immunotherapy. Obesity is likely to cause metabolic abnormalities such as hyperglycemia and hyperlipidemia, providing favorable conditions for the synthesis of tumor cell membranes, energy metabolism, and rapid proliferation, which further promotes the growth of tumors (24, 25). A decrease in muscle mass will affect the function of immune cells and the activation of stromal cells in the tumor microenvironment. The disruption of the immune balance weakens the attack of the immune system on tumor cells, promoting the proliferation, invasion, and metastasis, and inhibiting the function of immune cells (26–28).

An ECOG-PS ≥ 3 (*HR* = 5.274, 95% CI = 2.670–10.418) was also confirmed as an independent prognostic factor. Patients with an ECOG-PS score of ≥ 3 had a 5.274 times higher risk of OS than those with an ECOG-PS score of < 3. ECOG-PS ≥ 3 reflects an extremely poor physical condition, indicating a decline in overall body function. This deterioration makes it challenging for patients to tolerate the burden of tumors and immunotherapy, which negatively impacts survival. Multiple studies have shown that the lower the ECOG-PS score, the higher the survival rate of patients. Blagden et al. (29) conducted a study on NSCLC patients and found that, after adjusting for sex and disease stage, the ECOG-PS score was an independent predictor of survival in multivariate Cox regression analysis. Patients with an ECOG-PS score of 0–1 demonstrated significantly longer median survival compared to those with an ECOG-PS score of 2–4. Similarly, Ma et al. (30) analyzed 988 small cell lung cancer patients and observed that an ECOG-PS score of 0–1 was associated with better OS (median OS: 17 months for ECOG 0–1 vs. 11 months for an ECOG 2–3, *p* < 0.001).

Multivariable Cox regression analysis demonstrated that patients receiving later-line ICI therapy faced significantly elevated mortality rates compared to first-line recipients (2 L: *HR* = 4.274, 95% *CI*:1.941–9.411; ≥3 L: *HR* = 2.980, 95% *CI*:1.169–7.597). These findings are consistent with previously reported outcomes by Li S et al. (11). This pattern may be explained by the more preserved immune competence and higher tumor immunogenicity typically observed in treatment-naïve patients. In contrast, those progressing to later lines of treatment often present with a higher tumor burden and more aggressive disease biology, representing a population with intrinsically diminished therapeutic potential (31). While PD-L1 expression represents a well-established predictive biomarker for ICI response (32), its assessment was not included in our multivariate analysis due to limited data availability, highlighting the need for future studies incorporating standardized PD-L1 evaluation to further elucidate the relationship between treatment line and immunotherapy efficacy.

It is particularly worth noting that the results of the multivariate analysis show that the creatinine level is an independent prognostic factor (*HR* = 0.963, 95% *CI* = 0.936–0.990). This suggests that an increase in the creatinine level is actually associated with a reduced risk of mortality. The underlying mechanism of this phenomenon may be closely related to the metabolic characteristics of creatinine production. Creatinine is continuously produced by muscle tissue through the metabolism of phosphocreatine, and its production is positively correlated with muscle mass (33). In the SO group, there was a decrease in muscle mass, which resulted in a compensatory reduction in the rate of creatinine production. As a result, serum creatinine levels failed to accurately reflect the glomerular filtration function. Even in cases of renal function damage, the reduced generation of creatinine from muscle sources can cause the serum creatinine test values to remain within the normal range, thereby masking the true state of renal function. This complex relationship between creatinine production and renal function assessment suggests that relying solely on serum creatinine levels to judge renal function and prognosis in clinical practice has limitations. It is necessary to combine multiple indicators, such as muscle mass assessment and glomerular filtration rate, for a more accurate evaluation of patient prognosis.

Interestingly, our research findings indicate that obese patients with sarcopenia have the poorest prognosis. In multivariate analysis, isolated sarcopenia or obesity did not predict the survival rates, highlighting the synergistic effect of these conditions. In SO patients, there is usually no obvious short-term weight change or visible signs of weight loss, and they are often misdiagnosed as having no nutritional risk without a computerized tomography (CT) scan. In this study, our research shows that SO is a potential predictor of overall survival in lung cancer patients receiving ICIs and serves as an independent prognostic factor. Without timely nutritional interventions, malnutrition can have an adverse impact on the prognosis of patients and lead to serious consequences. Implementing appropriate detection can help achieve early discovery, early diagnosis, and timely nutritional intervention, which increases the possibility of improving clinical prognosis. Therefore, timely identification and screening of malnourished patients with SO are crucial for helping them reverse their malnutrition status (34, 35).

This study has several limitations. First, its retrospective design could not draw causal inferences, and the small sample size in the sarcopenic obesity subgroup (*n* = 18) may limit statistical power. Second, the single-center cohort and strict inclusion criteria may introduce selection bias. Third, the potential limitations of the CT-based body composition analysis, including technical variations in scanning protocols and patient-related factors, should be considered when interpreting the results. Additionally, the CT-based sarcopenia cutoff values were derived from Western populations, although the methodology was validated among Chinese lung cancer patients. Future prospective, multi-center studies with larger sample sizes are needed to validate these findings and establish optimized CT diagnostic thresholds specific to the Asian populations. Despite these limitations, our study provides robust preliminary evidence through rigorous body composition assessments, adjustments for key confounders, and long-term follow-up.

## 5 Conclusion

This study indicated that SO was independently associated with poorer survival outcomes, even after adjusting for clinical and laboratory confounding factors. This highlights the crucial interaction between muscle depletion and fat mass in determining prognosis during immunotherapy, serving as a valuable reference for assessing the prognosis of lung cancer patients, particularly those receiving immunotherapy. Incorporating body composition assessments into clinical practice could help improve risk stratification and facilitate more precise disease evaluation and personalized treatment planning.

## Data Availability

The original contributions presented in the study are included in the article material, further inquiries can be directed to the corresponding author.

## Footnotes

1
https://s.wzhealth.com/AIModelAnalysis/#/home?redirect=%2Findex

## References

[1] Sung H Ferlay J Siegel RL Laversanne M Soerjomataram I Jemal A . Global Cancer statistics 2020: GLOBOCAN estimates of incidence and mortality worldwide for 36 cancers in 185 countries. CA Cancer J Clin. (2021) 71:209–49. doi: 10.3322/caac.21660, 33538338

[2] Riudavets M de Garcia Herreros M Besse B Mezquita L. Radon and lung cancer: current trends and future perspectives. Cancers. (2022) 14:3142. doi: 10.3390/cancers14133142, 35804914 PMC9264880

[3] Lee CS Devoe CE Zhu X Fishbein JS Seetharamu N. Pretreatment nutritional status and response to checkpoint inhibitors in lung cancer. Lung Cancer Manag. (2020) 9:LMT31. doi: 10.2217/lmt-2020-0008, 32346405 PMC7186851

[4] Borghaei H Paz-Ares L Horn L Spigel DR Steins M Ready NE . Nivolumab versus docetaxel in advanced nonsquamous non-small-cell lung cancer. N Engl J Med. (2015) 373:1627–39. doi: 10.1056/NEJMoa1507643, 26412456 PMC5705936

[5] Guller M Herberg M Amin N Alkhatib H Maroun C Wu E . Nutritional status as a predictive biomarker for immunotherapy outcomes in advanced head and neck cancer. Cancers. (2021) 13:5772. doi: 10.3390/cancers13225772, 34830929 PMC8616447

[6] Cruz-Jentoft AJ Bahat G Bauer J Boirie Y Bruyere O Cederholm T . Sarcopenia: revised European consensus on definition and diagnosis. Age Ageing. (2019) 48:601. doi: 10.1093/ageing/afz046, 31081853 PMC6593317

[7] Veronese N Ragusa FS Pegreffi F Dominguez LJ Barbagallo M Zanetti M . Sarcopenic obesity and health outcomes: an umbrella review of systematic reviews with meta-analysis. J Cachexia Sarcopenia Muscle. (2024) 15:1264–74. doi: 10.1002/jcsm.13502, 38897937 PMC11294015

[8] Liu C Liu T Deng L Zhang Q Song M Shi J . Sarcopenic obesity and outcomes for patients with Cancer. JAMA Netw Open. (2024) 7:e2417115. doi: 10.1001/jamanetworkopen.2024.17115, 38874924 PMC11179127

[9] Gao Q Hu K Gao J Shang Y Mei F Zhao L . Prevalence and prognostic value of sarcopenic obesity in patients with cancer: a systematic review and meta-analysis. Nutrition. (2022) 101:111704. doi: 10.1016/j.nut.2022.111704, 35696740

[10] Martin L Birdsell L Macdonald N Reiman T Clandinin MT McCargar LJ . Cancer cachexia in the age of obesity: skeletal muscle depletion is a powerful prognostic factor, independent of body mass index. J Clin Oncol. (2013) 31:1539–47. doi: 10.1200/JCO.2012.45.2722, 23530101

[11] Li S Liu Z Ren Y Liu J Lv S He P . Sarcopenia was a poor prognostic predictor for patients with advanced lung cancer treated with immune checkpoint inhibitors. Front Nutr. (2022) 9:900823. doi: 10.3389/fnut.2022.900823, 35923193 PMC9339782

[12] Sahin MEH Akbas F Yardimci AH Sahin E. The effect of sarcopenia and sarcopenic obesity on survival in gastric cancer. BMC Cancer. (2023) 23:911. doi: 10.1186/s12885-023-11423-y, 37770828 PMC10537530

[13] Li Y Hou J Chen R. Prognostic value of sarcopenic visceral obesity in hepatocellular carcinoma treated with TACE. Medicine. (2023) 102:e34292. doi: 10.1097/MD.0000000000034292, 37417609 PMC10328579

[14] Bolte FJ McTavish S Wakefield N Shantzer L Hubbard C Krishnaraj A . Association of sarcopenia with survival in advanced NSCLC patients receiving concurrent immunotherapy and chemotherapy. Front Oncol. (2022) 12:986236. doi: 10.3389/fonc.2022.986236 36212442 PMC9539742

[15] Lin TY Chen YF Wu WT Han DS Tsai IC Chang KV . Impact of sarcopenia on the prognosis and treatment of lung cancer: an umbrella review. Discover Oncol. (2022) 13:115. doi: 10.1007/s12672-022-00576-0, 36307591 PMC9616989

[16] Khaddour K Gomez-Perez SL Jain N Patel JD Boumber Y. Obesity, sarcopenia, and outcomes in non-small cell lung cancer patients treated with immune checkpoint inhibitors and tyrosine kinase inhibitors. Front Oncol. (2020) 10:576314. doi: 10.3389/fonc.2020.576314, 33194687 PMC7607047

[17] Duggal NA Pollock RD Lazarus NR Harridge S Lord JM. Major features of immunesenescence, including reduced thymic output, are ameliorated by high levels of physical activity in adulthood. Aging Cell. (2018) 17:e12750. doi: 10.1111/acel.12750 29517845 PMC5847865

[18] Crane JD MacNeil LG Lally JS Ford RJ Bujak AL Brar IK . Exercise-stimulated interleukin-15 is controlled by AMPK and regulates skin metabolism and aging. Aging Cell. (2015) 14:625–34. doi: 10.1111/acel.12341, 25902870 PMC4531076

[19] Bachus H McLaughlin E Lewis C Papillion AM Benveniste EN Hill DD . IL-6 prevents Th2 cell polarization by promoting SOCS3-dependent suppression of IL-2 signaling. Cell Mol Immunol. (2023) 20:651–65. doi: 10.1038/s41423-023-01012-1, 37046042 PMC10229632

[20] Weisberg SP McCann D Desai M Rosenbaum M Leibel RL Ferrante AW Jr. Obesity is associated with macrophage accumulation in adipose tissue. J Clin Invest. (2003) 112:1796–808. doi: 10.1172/jci200319246, 14679176 PMC296995

[21] Baker RG Hayden MS Ghosh S. NF-κB, inflammation, and metabolic disease. Cell Metab. (2011) 13:11–22. doi: 10.1016/j.cmet.2010.12.008, 21195345 PMC3040418

[22] Yang J He J Feng Y Xiang M. Obesity contributes to hepatocellular carcinoma development via immunosuppressive microenvironment remodeling. Front Immunol. (2023) 14:1166440. doi: 10.3389/fimmu.2023.1166440, 37266440 PMC10231659

[23] Green WD Beck MA. Obesity altered T cell metabolism and the response to infection. Curr Opin Immunol. (2017) 46:1–7. doi: 10.1016/j.coi.2017.03.008, 28359913 PMC5554716

[24] Hopkins BD Goncalves MD Cantley LC. Obesity and cancer mechanisms: cancer metabolism. J Clin Oncol. (2016) 34:4277–83. doi: 10.1200/jco.2016.67.9712 27903152 PMC5562429

[25] Abdelaal M le Roux CW Docherty NG. Morbidity and mortality associated with obesity. Ann Transl Med. (2017) 5:161. doi: 10.21037/atm.2017.03.107 28480197 PMC5401682

[26] Kasprzak A. The role of tumor microenvironment cells in colorectal cancer (CRC) cachexia. Int J Mol Sci. (2021) 22:1565. doi: 10.3390/ijms22041565, 33557173 PMC7913937

[27] Shen L Zong Y Zhao J Yang Y Li L Li N . Characterizing the skeletal muscle immune microenvironment for sarcopenia: insights from transcriptome analysis and histological validation. Front Immunol. (2024) 15:1414387. doi: 10.3389/fimmu.2024.1414387, 39026669 PMC11254692

[28] Park SY Hwang BO Song NY. The role of myokines in cancer: crosstalk between skeletal muscle and tumor. BMB Rep. (2023) 56:365–73. doi: 10.5483/bmbrep.2023-0064, 37291054 PMC10390289

[29] Blagden SP Charman SC Sharples LD Magee LR Gilligan D. Performance status score: do patients and their oncologists agree? Br J Cancer. (2003) 89:1022–7. doi: 10.1038/sj.bjc.6601231, 12966419 PMC2376959

[30] Ma X Zhang Z Chen X Zhang J Nie J Da L . Prognostic factor analysis of patients with small cell lung cancer: real-world data from 988 patients. Thorac Cancer. (2021) 12:1841–50. doi: 10.1111/1759-7714.13846, 33955685 PMC8201544

[31] Reck M Rodríguez-Abreu D Robinson AG Hui R Csőszi T Fülöp A . Pembrolizumab versus chemotherapy for PD-L1-positive non-small-cell lung cancer. N Engl J Med. (2016) 375:1823–33. doi: 10.1056/NEJMoa1606774, 27718847

[32] Liu J Man Y Gao J Wang X Zhang L Li M . Correlation between PD-L1 expression status and efficacy of immunotherapy as second-line or later-line therapy in advanced non-small cell lung cancer patients. Eur J Cancer Prev. (2024) 33:448–60. doi: 10.1097/CEJ.0000000000000880, 38386588

[33] Groothof D Shehab NBN Erler NS Post A Kremer D Polinder-Bos HA . Creatinine, cystatin C, muscle mass, and mortality: findings from a primary and replication population-based cohort. J Cachexia Sarcopenia Muscle. (2024) 15:1528–38. doi: 10.1002/jcsm.13511, 38898741 PMC11294032

[34] Barazzoni R Gortan CG. Double burden of malnutrition in persons with obesity. Rev Endocr Metab Disord. (2020) 21:307–13. doi: 10.1007/s11154-020-09578-1, 32766943 PMC7455581

[35] Mwala NN Borkent JW van der Meij BS van der Schueren MAE. Challenges in identifying malnutrition in obesity; an overview of the state of the art and directions for future research. Nutr Res Rev. (2025) 38:219–28. doi: 10.1017/S095442242400012X 38576127 PMC7616526

